# Children's Strategy Choices on Complex Subtraction Problems: Individual Differences and Developmental Changes

**DOI:** 10.3389/fpsyg.2018.01209

**Published:** 2018-07-17

**Authors:** Sara Caviola, Irene C. Mammarella, Massimiliano Pastore, Jo-Anne LeFevre

**Affiliations:** ^1^Department of Psychology, University of Cambridge, Cambridge, United Kingdom; ^2^Department of Developmental Psychology, Università degli Studi di Padova, Padova, Italy; ^3^Department of Psychology, Institute of Cognitive Science, Carleton University, Ottawa, ON, Canada

**Keywords:** mental calculation, subtraction problems, strategy choice, children, mathematics, problem solving, arithmetic

## Abstract

We examined how children's strategy choices in solving complex subtraction problems are related to grade and to variations in problem complexity. In two studies, third- and fifth-grade children (N≈160 each study) solved multi-digit subtraction problems (e.g., 34–18) and described their solution strategies. In the first experiment, strategy selection was investigated by means of a *free-choice* paradigm, whereas in the second study a *discrete-choice* approach was implemented. In both experiments, analyses of strategy repertoire indicated that third-grade children were more likely to report less-efficient strategies (i.e., counting) and relied more on the right-to-left solution algorithm compared to fifth-grade children who more often used efficient memory-based retrieval and conceptually-based left-to-right (i.e., decomposition) strategies. Nevertheless, all strategies were reported or selected by both older and younger children and strategy use varied with problem complexity and presentation format for both age groups. These results supported the overlapping waves model of strategy development and provide detailed information about patterns of strategy choice on complex subtraction problems.

## Introduction

Understanding how children choose and apply a specific strategy to solve a mathematical problem is an important issue in the field of numerical cognition. Individuals' strategy choices are influenced by many factors, including their repertoire or knowledge of strategies (Kilpatrick et al., 2001; Baroody and Dowker, 2003), their expertise in implementing those strategies (Torbeyns et al., 2006; Verschaffel et al., 2007), and their overall level of mathematical achievement (Geary et al., 2000; Smith-Chant and LeFevre, 2003). Although some studies have examined children's performance in solving complex arithmetic problems, the results have varied depending on children's age and consequently their arithmetic expertise, the specific arithmetic operation, and the type of strategy assessment applied (e.g., Torbeyns et al., 2009a,b; Torbeyns and Verschaffel, 2016; Lemaire and Brun, 2018). Accordingly, in the present research we conducted two large scale studies using two different types of strategy assessment in which we investigated how children at different stages of their primary education perform complex subtraction problems varying in the degree of complexity. By manipulating these key variables, we were able to provide a comprehensive overview of the factors influencing children's strategy choices on multi-digit subtraction problems.

Among the four basic arithmetic operations, that is, addition, subtraction, multiplication, and division, subtraction is of particular interest because children and adults report using a wide range of different strategies, both on simple problems such as 14–6 (e.g., LeFevre et al., 2006) and on complex problems such as 24–9 or 56–23 (Torbeyns et al., 2009a; Linsen et al., 2014). According to Lemaire and Siegler (1995), arithmetical strategy use entails two components: first choosing a strategy (*strategy choice*), and then implementing the chosen strategy (*strategy execution*) to solve the arithmetic problem. In the present study, the focus was on the process of strategy choice. Strategy choice is associated not only with an individual's level of skill in simple arithmetic (Smith-Chant and LeFevre, 2003), but also with the specific features of a given problem (e.g., problem difficulties in terms of number of digits in each operand or the presence/absence of a borrow procedure; Imbo et al., 2007), and the context of the problem, such as the presentation format (e.g., problems presented in horizontal vs. vertical format Trbovich and LeFevre, 2003; Lemaire and Calliès, 2009; Imbo and LeFevre, 2010). Like Lemaire and Brun (2018), in the present study we provide a detailed analysis of strategies used by children to solve subtraction problems, investigating for the first time how different problem features (i.e., problem complexity and presentation format together) influence children's strategies and performance, as well as how such strategic behavior changes with children's age and related expertise.

### Subtraction strategies

Adults and children have been observed to use a variety of strategies on subtraction problems and these strategies can be categorized according to the type of computations involved. For the very simplest problems, such as 5–3, memory retrieval is usually reported, but various counting strategies, such as counting up (i.e., for 5–3, counting 4, 5) are also used (e.g., LeFevre et al., 2006). Solvers may also report counting up or down on large problems such as 52–49 (Torbeyns et al., 2009b). Another way of categorizing strategies on more complex problems is to consider the solution path, such that strategies can be divided into two main categories (Green et al., 2007; Imbo and LeFevre, 2009). In *right-to-left* strategies, the operands are treated as concatenations of single digits and calculation considers each columnar operation separately, for example, solving 45–29 by subtracting 15–9 = 6 and 3–2 = 1. For subtraction, the right-to-left strategy is a mental version of column-by-column algorithm taught at school for use in written calculation. In contrast, in *left-to-right* strategies, operands are represented and manipulated in a more holistic manner. For example, 64–12 can be decomposed into 60–10 = 50 and 4–2 = 2, and then reassembled to obtain the answer (i.e., 52; Lemaire and Calliès, 2009), or 69–13 can be solved by rounding to 70–13 = 57 and then subtracting 1. Often referred to as transformation or decomposition strategies, the left-to-right approach requires conceptual understanding of the structure of numbers (LeFevre et al., 2006).

Children show developmental and educational changes in the use of the left-to-right and right-to-left strategies described above, mainly corresponding to strengthening of their mental calculation skills due to the acquisition of multi-digit algorithms (written) procedures (Fuson et al., 1988; Geary et al., 2004). In particular, in European countries such as Belgium, Italy, and the Netherlands, children are taught mental computation for multi-digit subtraction in second grade, and start to learn the written algorithm in third grade (Cornoldi and Lucangeli, 2004; Caviola et al., 2014). However, in the US and Canada, less emphasis is placed on mental computation, especially for multi-digit problems, which are thought to be solved through the written algorithm (Baroody and Dowker, 2003). Accordingly, strategy use might vary across countries and school systems.

A few researchers have studied children's strategies on complex subtraction but none has documented the full range of strategies used (Beishuizen, 1993; Lemaire and Calliès, 2009). For example, Lemaire and Calliès (2009) compared the performance of 20 fifth- and 20 seventh-grade students in France on complex subtractions, however, they restricted children's strategy choices to two left-to-right methods (i.e., full and partial decomposition). Other researchers have focused on subtraction-by-addition strategies (e.g., Linsen et al., 2014). Thus, currently there is no information about the extent of children's spontaneous strategy use on complex subtraction.

Other important factors in children's strategy choices, beyond their levels of automaticity and strategy knowledge, are the characteristics of the problem. Problem complexity is one feature that is assumed to influence children's strategy choices (Lemaire and Lecacheur, 2011; Ardiale and Lemaire, 2013). One way to vary problem complexity is to include a carry or borrow requirement (Noël et al., 2001; Imbo et al., 2007). Superficial features of the problems, such as presenting the problems in a horizontal vs. a vertical format, may also influence children's strategy choice. Overall, processing efficiency varies with presentation format, and complexity (Trbovich and LeFevre, 2003; Lemaire and Calliès, 2009; Imbo and LeFevre, 2010; Caviola et al., 2012), but, to the best of our knowledge, none of the previous studies examined in a larger set of problems how different features can interact to influence children's strategy choice. Moreover, researchers have suggested that manipulations of presentation format can trigger differential recruitment of cognitive resources, leading to variability in the solution procedures that participants select as a function of format (Trbovich and LeFevre, 2003). For example, vertically-presented problems required more visual resources, whereas horizontally-presented problems required more phonological resources (Caviola et al., 2012). Thus, presentation format may influence selection of strategies, however, this possibility has not been assessed in children.

### Methods for studying strategy choices

In the strategy literature, among others, the two most widely used methods implemented to evaluate strategy choice and execution are *free-choice* report and *forced-choice* methods. Free-choice reports require participants to verbally report which strategy they used to solve a problem immediately after solving it with no restrictions on the strategy repertoire (e.g., Siegler, 1988; Davis and Carr, 2001; Mabbott and Bisanz, 2003; Noël et al., 2004; Torbeyns et al., 2005). This method leads to a broader collection of strategies than forced-choice methods, under the assumption that participants have sufficient metacognitive/introspective and verbal abilities enabling them to provide accurate verbal report of strategies used (Kirk and Ashcraft, 2001). Free-choice reports may not be valid for processes that are fast and automatic (Ericsson and Simon, 1993), and may not be appropriate for children if their verbal abilities are limited (Siegler and Stern, 1998). On this view, free-choice reports may potentially lead to biases, such as the over-reporting of strategies whose salience is high, and conversely, the under-reporting of fast or less procedural ones (Ericsson and Simon, 1993; Kirk and Ashcraft, 2001; Thevenot et al., 2010).

To address some of the limitations of free-choice retrospective reports, Siegler and Lemaire (1997) developed a modified method, known as the choice/no-choice paradigm. This method consists of a forced choice condition, where individuals apply a preferred strategy (chosen from a restricted set of two or three options), and two or more no-choice conditions, where they are required to solve all problems with a single strategy; thus, the number of no-choice conditions corresponds to the number of options in the forced-choice condition (e.g., Imbo and Vandierendonck, 2007a,b,c; Reed et al., 2015). The choice/no-choice paradigm provides information on strategy *efficiency* from the no-choice condition, independently of the choice process, whereas the comparison of performance in the no-choice and the choice condition gives an indication of people's strategy *adaptivity* (i.e., the selection of the most efficient procedure from the limited set provided). Although this method does address some of the problems of the free choice approach, specifically, the concern that strategy choice and efficiency are confounded, there are also criticisms (Luwel et al., 2009). In particular, the choice/no-choice approach limits the strategies that are available because a limited number of no-choice conditions are included and thus may not provide the “best” strategy on any given trial (cf. Imbo and LeFevre, 2011; Xu et al., 2014). In order to address these limitations, some authors have implemented a method that we can define as a *discrete-choice method*, where participants were asked to select between a larger number of given strategy alternatives, within the prospective that providing a broader choice of strategies gives better information about children's choices (e.g., Lemaire and Brun, 2018). The strength of this *discrete-choice* approach lies in the opportunity to determine the effects of explanatory variables from a more extensive set of options, which provides less restriction on individuals' strategy selection (Xu et al., 2014). A similar *discrete-choice* strategy method, in which a set of options was provided, has been used extensively within the domain of simple arithmetic with adults (e.g., Campbell and Xue, 2001; Campbell and Austin, 2002; Imbo and Vandierendonck, 2007a, 2008) and with children, combined with a choice/no-choice experimental design (Imbo and Vandierendonck, 2007b).

In the present study, rather than focusing solely on one single method to assess strategy choice, we tested two cohorts of third- and fifth-school graders in two different experiments in order to indirectly compare free-choice and a discrete-choice approaches.

### The present research

The central questions in the present research were how children's strategy choices are influenced by (a) their level of expertise (e.g., grade 3 vs. 5), (b) problem features (e.g., complexity and contextual features), and (c) the type of strategy assessment to collect strategy reports. In two experiments, children in the same age-range were tested with the same pool of multi-digit subtraction problems (e.g., 23–3; 47–19). Across experiments, two methods of assessing children's strategy choice were used. In Experiment 1, strategies were assessed on a problem-by-problem basis by means of immediate retrospective verbal self-reports. In Experiment 2, children were asked to choose the strategies used from among a list of alternatives that was based on the solution procedures observed in Experiment 1.

In order to assess the role of expertise in strategy choices, we tested Italian children in grades three and five. This age range is assumed to cover an important transition period between the use of mental and written strategies: children in third grade have yet to fully master multi-digit calculation, but by fifth grade they will have started to automatize more efficient calculation skills (Baroody and Dowker, 2003). In particular, Italian curriculum for teaching arithmetic is based on the written standard approach that requires children in grade 1 (typically 6 years old) to consolidate their counting skills and start learning the principles of adding and subtracting (*left-to-right* strategies). In second grade, the procedures for solving written additions and subtractions calculation are taught (in that order) using a columnar (*right-to-left*) strategy (Cornoldi and Lucangeli, 2004). Thus, third-grade children are expected to be reasonably skilled at single-digit computations and are presumably more likely to use simpler but less efficient strategies (e.g., counting) compared to fifth-grade children. In contrast, fifth-grade children are expected to have more efficient calculation skills —they should be more likely to accurately retrieve arithmetic facts and to have a greater knowledge of efficient strategies such as decomposition or the right-to-left algorithm (Baroody and Dowker, 2003). Thus, large differences in strategy selection could be anticipated in this age range (cf. Lemaire and Calliès, 2009; Lemaire and Brun, 2018).

Problem features also expected to influence the choice of strategy: Children are more likely to use memory retrieval for easier problems when this strategy will probably produce the right answer, whereas they choose computational strategies for more difficult problems when retrieval is less likely to generate the right answer (Siegler, 1996; Lemaire and Calliès, 2009). The likelihood of a given computational strategy being chosen thus depends on the characteristics of the problem. There is evidence to suggest that children are unlikely to use the most advanced computational strategy available to them unless the difficulty of the problem demands it. On this view, increasing the difficulty of the problem will promote the use of more advanced computational strategies because children will maximize efficiency while preserving accuracy. Efficiency usually declines for problems that involve carrying or borrowing (Noël et al., 2001; Imbo et al., 2007), as well as for problems with increasing number of digits (Green et al., 2007; Imbo et al., 2007). Thus, to explore how problem features influenced strategy choices for these children, we manipulated the complexity of the problems (i.e., whether a borrow was required and the number of digits in the problem) and presentation format (i.e., horizontal vs. vertical).

In summary, the goals of the present experiments were to (a) compare two different methods to assess children' strategy choice; (b) replicate previous findings on how children' age effects performance in solving complex subtraction problems; and (c) test the relations among children' age, problem features, and strategy choice. Further, we used a novel method to analyze strategy choices, specifically, multinomial modeling of the frequency of strategy choices. Accordingly, our emphasis was not on strategy adaptivity (which is the focus of the choice-no-choice method); instead, we explored the development of strategy choices according children's own repertoire.

Overall, we expected to find similar patterns in both experiments. First, we predicted that fifth-graders would make more use of retrieval and less use of counting than third-graders in solving subtractions without borrowing (e.g., 24–3). Second, we expected to see an increasingly efficient use of the decomposition strategy by older children, and a more consistent use of the right-to-left strategy, especially in problems presented in columns with borrowing. Third, we expected that children would perform better while solving single-digit no-borrow problems (e.g., 45–2) than while solving double-digit borrow problems (e.g., 45–19) and would use decompositions (left-to-right procedures) and standard algorithms (right-to-left procedures) more on complex problems than on simpler problems. Finally, we predicted that children would be more likely to solve horizontally-presented problems with decomposition strategies and vertically presented problems with the right-to-left algorithm (Trbovich and LeFevre, 2003; DeStefano and LeFevre, 2004).

## Experiment 1

In this study, we tested children's strategy selection on multi-digit subtraction problems by means of immediate retrospective self-report. In addition to replicating the results of previous studies on complex subtraction (Torbeyns and Verschaffel, 2016; Lemaire and Brun, 2018), we wanted to (a) determine the full repertoire of strategies used by children in solving this type of problem, and (b) analyze the mediating roles of children's age and problem features on strategy choice.

## Methods

### Participants

Participants included 155 children: 76 in third grade (50 boys, 26 girls) with a mean age of 105.9 months (*SD* = 3.8; range = 99–112 months), and 79 in fifth-grade (42 boys, 37 girls) with a mean age of 129.8 months (*SD* = 3.5; range = 124–143 months) who were attending Italian urban state schools. Parental consent was obtained. Children with special educational needs, intellectual disabilities, or neurological/genetic disorders, as indicated by their teachers, were not included in the study.

### Materials

#### Arithmetical achievement

To assess arithmetical achievement, participants were initially presented with paper-and-pencil tasks adapted from an age-standardized Italian battery (Biancardi and Nicoletti, 2004). In the c*omplex written calculation* test, children attempted 12 written calculation problems (4 additions, 4 subtractions, and 4 multiplications; e.g., 46+18 = ?; 54–27 = ?; 23 × 41 = ?) without time limits. Scores were total correct (Cronbach's alpha = 0.78). In the s*imple calculation* test, children attempted 16 problems (8 additions and 8 subtractions) with operands between 1 and 9. For both addition and subtraction half of the results were less than 10 (e.g., 4+2 = ?; 7−5 = ?), and the other half were more than 10 (e.g., 10+12 = ?; 30–6 = ?). The total time allowed to complete the test was 200 s. Cronbach's reliability coefficients were higher than 0.80 for each set of problems.

#### Computer-based experimental task

Children solved multi-digit subtraction problems. Two problem sets were created, each with 32 problems (see the Supplementary Material for the whole sets). In order to manipulate problem difficulty, problems were characterized by the presence/absence of borrowing procedure and by the number of digit of the subtrahend. One set required a borrow procedure in the unit position (e.g., 31–19 = ?), and the other set did not require a borrow procedure (e.g., 38–26 = ?). Half of each set had a subtrahend with a one-digit number (e.g., 58–6) and the other half had a subtrahend with a two-digit number (e.g., 43–12). The correct answers for all the 64 subtraction problems ranged from 11 to 62. Following previous literature, to control the difficulty of the individual problems, certain types were excluded (e.g. Campbell, 2005): (a) no operand had 0 or 5 as the unit digit; (b) digits were not repeated in the same decade or unit positions across operands (e.g., 64−24 = ?); (c) no digits were repeated within operands (e.g., 66−31 = ?); (d) no correct answers for the decades or units equaled 0 (e.g., 36−16 = ?); and, finally, (e) no correct answers coincided with the second operand (e.g. 24−12 = ?). Furthermore, the outcome of subtractions (i.e., odd or even numbers) and the presentation format (i.e. horizontal or vertical) were controlled. Within each set, half of the problems were assigned to the vertical presentation and half to the horizontal presentation.

#### Procedure

Children were tested in two sessions. At the beginning of the experimental session, in a group session lasting about 30 min, individuals' mathematical achievement was assessed with paper-and-pencil tasks adapted from the standardized Italian battery developed by Biancardi and Nicoletti (2004) in their classroom. In an individual session lasting ~60 min, the children were tested in a quiet room using the computer-based experimental task. The task was programmed using E-Prime software (Psychology Software Tools, Inc., Pittsburgh, PA, USA) and presented on a 15-inch 1024 × 768 pixel computer screen. Problems were shown in 72-point Times New Roman black font on a white background in the center of the screen. Participants sat 60 cm from the screen. The E-Prime software controlled how long time the stimulus was displayed and recorded accuracy, response times (RTs), and the selected strategies for each trial.

Each trial began with the presentation of a fixation point (^*^) in the center of the computer screen for 750 ms. Then a problem was displayed (horizontally or vertically) in the center of the screen. Each trial was timed as of the moment when the problem appeared on the screen and ended when the experimenter pressed a button as promptly as possible after participants gave their answers. Problems were presented in a pseudo-random order.

Children were randomly assigned to one of two problem sets, that is, problems with or without a borrow requirement, such that there were 73 children (39 boys, 34 girls; 37 third- and 36 fifth-graders) in the no-borrow condition, and 82 (53 boys, 29 girls; 39 third-, and 43 fifth-graders) in the borrow condition.

Each participant solved 4 practice trials and 32 experimental trials. Trial-by-trial feedback on calculation accuracy was only given during the practice trials. Children were told that they would see two-digit subtraction problems (e.g., 79-37; 92-59) on the computer screen: they were asked to do the calculation aloud and to give their answers aloud, focusing equally on speed and accuracy. Immediately after having provided each solution, they were asked to verbally explain how they had reached the result (each answer was recorded).

#### Classification of self-reports

Participants' verbal self-reports were classified into five different strategy categories by two trained judges on the basis of the narrative procedure descriptions. The two judges agreed on the classification of 97% of the problems. Five main strategies emerged when children's self-reports were analyzed. Trials were categorized as: (1) *retrieval* when participants simply reported remembering or knowing the answer (); (2) *counting* when children described a sequential subtraction of a one unit at a time (e.g., 24–3 as 24, 23, 22, answer 21); (3) *left-to-right decomposition* when the answers were obtained by breaking a larger problem down into smaller ones (e.g., regrouping strategies); (4) *right-to-left algorithm* when children described arriving at the answer by first subtracting the units and then the tens (e.g., …); (5) *other* when children reported guessing or a mixture of different procedures on the same problem.

## Results

### Arithmetical achievement

Performance on the two arithmetic achievement tasks was analyzed in 2 (grade) × 2 (condition: borrow, no-borrow) ANOVAs. Consistent with the use of a grade-standardized score, there were no effects of grade, and no effects of condition, indicating that children at both grades had mathematical abilities expected for their age, and that the two randomly assigned groups of children (borrow and no-borrow conditions) were equally matched on arithmetic skills. The descriptive statistics and ANOVAs results for these analyses are presented in the Supplementary Material.

### Accuracy and response times

Accuracy was the percentage of correct responses; response times were calculated on the basis of correct trials only.

The descriptive statistics for performance on the multi-digit subtraction task are shown in Table 1 (upper panel). In order to verify which manipulated variables influenced the performance of multi-digit subtraction problems, response times, and accuracy were analyzed in separate 2 (complexity: one- vs. two-digit numbers in the subtrahend) by 2 (format: horizontal, vertical presentation) × 2 (grade: 3, 5), × 2 (condition: borrow, no-borrow) mixed ANOVAs, with repeated measures on the first two factors. The results of these analyses are shown in Table 2. For the sake of simplicity, the two-way interactions are discussed only when the three-way interactions were not significant.

**Table 1:** Descriptive statistics (*M* = mean; *SD* = standard deviations) of multi-digit subtraction problems.

**Experiment 1**									
		**Third grade**				**Fifth grade**			
		**Horizontal**		**Vertical**		**Horizontal**		**Vertical**	
**Group**	**Complexity**	***M***	***SD***	***M***	***SD***	***M***	***SD***	***M***	***SD***
**ACCURACY**									
No-borrow	One-digit	92.91	10.84	92.22	12.62	95.83	6.68	95.13	9.57
	Two-digit	82.43	18.97	81.08	18.31	93.40	8.70	96.53	6.42
Borrow	One-digit	89.10	13.51	87.50	13.14	90.41	13.59	89.83	13.15
	Two-digit	69.55	27.48	79.16	20.75	71.80	23.64	82.56	16.62
**RESPONSE TIME**									
No-borrow	One-digit	8.73	4.03	8.96	4.17	3.66	1.07	4.03	1.34
	Two-digit	15.92	7.32	14.21	4.38	6.84	2.23	6.80	2.13
Borrow	One-digit	18.01	12.95	20.04	11.41	11.45	6.52	13.07	5.98
	Two-digit	26.34	15.51	23.92	12.57	21.21	16.31	17.93	7.99
**Experiment 2**									
**ACCURACY**									
No-borrow	One-digit	92.39	16.77	90.22	16.23	94.05	11.79	94.64	10.01
	Two-digit	79.62	23.33	77.17	22.25	85.12	20.52	84.23	16.35
Borrow	One-digit	72.87	27.23	74.39	26.21	83.42	21.25	81.79	22.00
	Two-digit	46.65	32.12	52.74	32.66	65.22	31.61	68.48	24.96
**RESPONSE TIME**									
No-borrow	One-digit	6.82	3.51	7.60	4.70	5.28	4.71	5.46	4.72
	Two-digit	14.15	5.65	13.38	4.48	10.65	7.92	9.89	6.11
Borrow	One-digit	15.82	7.51	17.41	7.93	11.73	6.28	12.70	6.33
	Two-digit	25.17	9.93	22.60	9.20	18.79	10.30	16.41	7.05

*Accuracy refers to the percentage of correct problems; response times are expressed in seconds*.

**Table 2:** Results of the mixed-design 2 × 2 × 2 × 2 ANOVAs for the accuracy and RTs, with grade (third and fifth grade) and condition (absence or presence of borrowing procedure) as the between-participants factors, and complexity (single or double-digit subtrahend) and format (horizontal or vertical presentation) as repeated measures (Experiment 1).

	**Accuracy**				**Reaction times**		
	***df***	***F***	***p***	***LogBF***	***F***	***p***	***LogBF***
Grade (G)	1,151	8.44^**^	0.004	13.12	28.83^**^	< 0.0001	40.43
Condition (Cond)	1,151	23.75^**^	< 0.0001	34.84	74.93^**^	< 0.0001	93.18
Complexity (C)	1,151	55.32^**^	< 0.0001	73.45	125.88^**^	< 0.0001	140.15
Format (F)	1,151	6.03^*^	0.015	9.16	1.14	0.288	1.74
G^*^Cond	1,151	2.58	0.110	4.18	0.06	0.802	0.10
G^*^C	1,151	4.84^*^	0.029	7.45	1.10	0.296	1.68
G^*^F	1,151	0.76	0.383	1.17	0.03	0.869	0.04
Cond^*^C	1,151	9.16^**^	0.003	13.90	4.60^*^	0.034	6.95
Cond^*^F	1,151	5.52^*^	0.020	8.52	0.09	0.765	0.14
C^*^F	1,151	12.75^**^	< 0.0001	18.95	11.62^**^	0.001	17.15
G^*^Cond^*^C	1,151	3.26	0.073	5.06	5.11	0.025	7.70
G^*^Cond^*^F	1,151	0.092	0.762	0.14	1.04	0.310	1.59
G^*^C^*^F	1,151	0.41	0.522	0.61	0.11	0.746	0.16
Cond^*^C^*^F	1,151	7.27^*^	0.008	11.20	4.13^*^	0.044	6.25
G^*^Cond^*^C^*^F	1,151	0.37	0.544	0.55	0.34	0.562	0.52

*LogBF, approximated bayes factor; G, Grade; Cond, Condition; C, Complexity; F, presentation Format*.

* *p < 0.05*;

** *p < 0.01*.

As expected, the main effect of grade was significant, showing that children in third grade performed significantly worse than those in fifth grade (84 vs. 89%). There were also main effects of format, condition and complexity: children showed a better performance when problems were vertically presented (88 vs. 85%), they solved borrow problems less accurately than no-borrow problems (82 vs. 91%), and they performed less accurately on problems with double-digit subtrahends than on those with single-digit subtrahends (82 vs. 92%). These differences were confirmed also by the two-way interaction between condition and complexity: the difference between borrow and no-borrow problems was larger for problems with double-digit subtrahends (i.e., 13%; 75% vs. 88%) than for problems with single-digit subtrahends (i.e., 5%; 89% vs. 94%), although both differences were significant (*p*_*s*_ < 0.001). The significant interaction between complexity and grade indicated that the difference among grades was due to the complexity of the problems: younger children registered lower performance only when they solved subtractions with double-digit subtrahends (i.e., 78 vs. 86%; *p* < 0.01).

Finally, the interaction between complexity × format and between condition × format were significant, as well as the three-way interaction among complexity, condition, and format. These interactions revealed that the presentation format influenced children's performance only when they were asked to solve the hardest problems, that is, double-digit subtrahends involving borrowing (i.e., 71 vs. 81%; *p* < 0.001).

**Figure 1:**
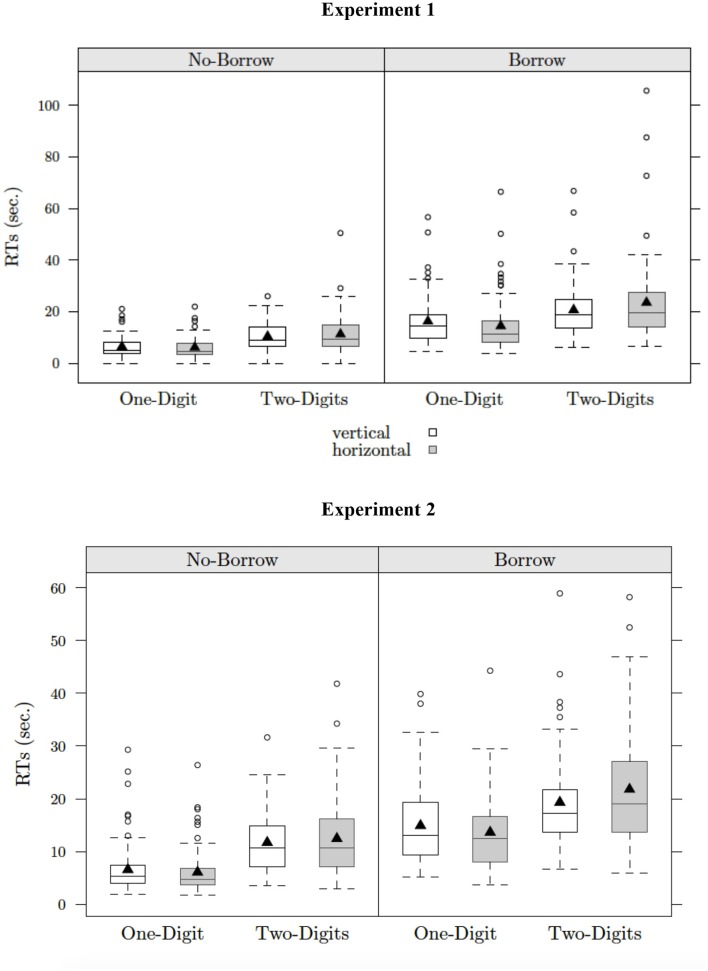
Representation of the three-way interaction of borrow^*^complexity^*^presentation format on correct RTs for Experiment 1 and 2.

The analysis of response time showed significant main effects of grade, condition (borrow status), and complexity. Hence, third-graders were slower than fifth graders (17 vs. 10 s), children who solved borrow problems responded more slowly than those who solved no-borrow problems (19 vs. 9 s), and children solved problems with double-digit subtrahends more slowly than those with single-digit subtrahends (17 vs. 11 s), as highlighted by the interaction of condition × complexity (*p*_s_ < 0.001). The complexity × format interaction was also significant. For problems with two-digit subtrahends, children solved problems in vertical format faster than those in horizontal format (16 vs. 18 s, *p* < 0.01) whereas, for problems with single-digit subtrahends, they solved problems in vertical format more slowly than those in horizontal format (12 vs. 10 s, *p* < 0.05). Finally, the three-way interaction among condition, complexity, and format is shown in Figure 1 (upper panel). Children who solved no-borrow problems did not show any effects of format, whereas those who solved borrow problems showed the interaction of complexity and format (*p*_s_ < 0.01).

### Self-report

The analyses of accuracy and response times showed that problem features influenced children's performance, and thus their strategy efficiency. Descriptive data on strategy choices for children in third- and fifth-grade are presented in Table 3 which shows the number of children who used the strategy at least once and the observed frequencies across grades, conditions, complexity, and presentation format.

**Table 3:** Descriptive data on strategy choices for children in third- and fifth-grade; showing the number of children who used the strategy at least once (*n*), the range, and the observed frequencies according grade, condition, complexity, and presentation format.

			**Third graders**										**Fifth graders**							
			**No borrow**				**Borrow**						**No borrow**				**Borrow**			
			**Simple**		**Complex**		**Simple**		**Complex**				**Simple**		**Complex**		**Simple**		**Complex**	
	***n***	**Range**	**Col**	**Row**	**Col**	**Row**	**Col**	**Row**	**Col**	**Row**	***n***	**Range**	**Col**	**Row**	**Col**	**Row**	**Col**	**Row**	**Col**	**Row**
**Experiment 1**	(*n* = 76)										(*n* = 79)									
Retrieval	29	0–14	90	94	0	1	1	1	1	0	38	0–18	188	192	5	7	2	0	2	1
Counting	31	0–23	47	50	2	3	28	29	10	17	28	0–16	27	28	5	0	18	24	1	2
Left-to-right	25	0–32	6	4	6	6	60	83	40	38	48	0–32	30	31	42	52	165	182	117	137
Right-to-Left	71	0–32	138	143	282	6	147	121	217	38	61	0–32	35	34	230	52	86	78	177	137
Other	34	0–32	15	5	6	7	76	78	44	52	34	0–30	8	3	6	3	73	60	47	45
**Experiment 2**	(*n* = 88)										(*n* = 87)									
Retrieval	39	0-19	59	74	27	20	28	29	12	14	70	0–27	171	181	48	40	60	86	24	25
Counting	76	0-32	121	127	98	95	115	109	59	56	59	0–30	40	44	43	35	81	77	71	52
Left-to-right	42	0-25	28	22	30	27	61	53	64	60	59	0–26	13	28	49	65	79	77	101	129
Right-to-Left	86	0-32	168	153	221	234	124	137	193	198	78	0–32	104	75	188	188	148	128	172	162

*n is the number of individuals in each grade who reported using the procedure at least once. Col, columnar (vertical) presentation; Row, horizontal presentation*.

The Table shows the number of children who reported using each strategy at least once: the use of strategies varied significantly across ages, in particular for the more complex strategies: more older children reported left-to-right strategy than younger children (61 vs. 33%), χ^2^ (1, *N* = 155) = 12.07, *p* < 0.001, Cramer's phi = 0.279 whereas younger children reported to use more often the right-to-left-algorithm (93 vs. 93%), χ^2^ (1, *N* = 155) = 8.05, *p* = 0.005, Cramer's phi = −0.228. For the simpler strategies, the differences are less evident, but the emerged pattern seems to indicate that older children are more likely to report retrieval and less likely to use counting compared to the younger children. Thus, the overall comparison of strategy repertoire across grade shows changes as a function of children's expertise: these shifts in strategy repertoire with grade are consistent with increased access to stored arithmetic facts and a greater conceptual understanding of number.

Next, we explore the patterns of strategy selection in relation to problem features. We analyzed strategy choices in order to determine whether they varied with the same problem features as did strategy efficiency. Of interest was the frequency of strategy choice across all problems, regardless of whether those choices resulted in accurate performance. As previously mentioned, the 155 children each solved 32 subtraction problems, hence there were a total of 4,960 trials for analysis.

Analyses of strategy choices were performed using the statistical software R (R Core Team, 2015) using the following packages: *vglm* for the Multinomial models (Yee and Wild, 1996; Yee, 2015) and *Bayes Factor* for Bayesian estimates (Morey and Rouder, 2015). To determine the best fitting model for describing the relation between problem features and strategy choices, we analyzed the data with a series of multinomial models. Each model included the four independent variables: grade, condition, complexity, and presentation format. This type of discrete-choice model permits the set of choice (the four strategy options) to vary by participants and can incorporate explanatory variables that can characterize the pattern of frequencies, in this situation, strategy choice.

A model-selection strategy was performed using a procedure to detect the best-fitting model (for an example, see Fox, 2015). The type of strategy that was selected on each trial was the dependent variable and there were four predictors: school grade attended (grade, with two levels: third and fifth grade); presence or absence of borrowing procedure (condition, with two levels: with or without borrowing); complexity of the subtrahend (subtrahend with one- or two-digits) and stimulus presentation format (two levels: horizontal or vertical). Then, starting from the null model (M0–i.e., the model including only intercepts and no predictors), we built the various models developed from all the possible combinations of the four predictors. After the null model (M0), we first explored the additive model; next all the possible two-way interactions were tested. Afterward, all the three-way interactions were explored. In total there were 14 models resulting from all the possible combination of the predictors—the saturated model with all predictors did not converge and so it was not included.

We used the likelihood ratio test to compare models, taking into consideration the Bayesian information criterion (BIC; Schwarz, 1978). In Table 3, the results of model comparisons are reported. ΔBIC indicates the differences between the null model (M0) and each subsequent model; a positive ΔBIC value implies that a given model is better than the null model. In order to compare the relative evidence for each different model we calculated the *Log Bayes Factor* (BF) approximations (see Table 4), using the formula (ΔBIC/2; Raftery, 1995). For example, a *Log BF* value of 3 indicates that one model is twenty [*exp* (3) = 20] times more likely than the null model, a difference that has been characterized as strong (Wagenmakers, 2007; Wetzels et al., 2011). More generally, the higher the ΔBIC and BF, the more likely the model is in comparison to the null model and thus provides a good fit to the data. Details of the multinomial process and the indexes that guided the model selection are given in Table 4.

**Table 4:** Model comparison for strategy choice in Experiment 1.

	***BIC***	***Δ_*BIC*_***	***Log BF***	**Model**
M0	3832	0		1
M1	468	3364	37.31	pres.form + condition + grade + complexity
M2	445	3387	25.68	complexity + condition ^*^ grade + pres.form
M3	460	3371	33.40	pres.form + condition + grade ^*^ complexity
M4	478	3353	42.44	grade ^*^ pres.form + condition + complexity
M5	422	3410	14.31	pres.form + grade + complexity ^*^ condition
M6	470	3362	38.03	grade + condition ^*^ pres.form + complexity
M7	478	3354	42.29	condition + grade + pres.form ^*^ complexity
M8	432	3399	19.45	grade ^*^ pres.form + condition ^*^ complexity
M9	462	3370	34.13	condition ^*^ pres.form + complexity ^*^ grade
M10	455	3377	30.67	complexity ^*^ pres.form + grade ^*^ condition
M11	393	3438		condition ^*^ grade ^*^ complexity + pres.form
M12	466	3366	36.25	complexity + condition ^*^ pres.form ^*^ grade
M13	486	3346	46.20	condition + pres.form ^*^ grade ^*^ complexity
M14	439	3393	22.82	grade + condition ^*^ complexity ^*^ pres.form

*BIC, Bayesian Information Criterion; Δ_BIC_, BIC difference with respect to the null model (M0); LogBF, approximated bayes factor respect to the best model (M1) calculated through the relative likelihood [i.e., (Δ_BIC_/2)]. The higher the Δ_BIC_ the better the model. Strategy, type of strategy (i.e., counting, retrieval, decomposition, and written calculation); Grade, 3rd and 5th grade; Complexity: single or double-digit subtrahend; Condition (absence or presence of borrowing procedure); Pres.form, vertical and horizontal presentation format*.

**Figure 2:**
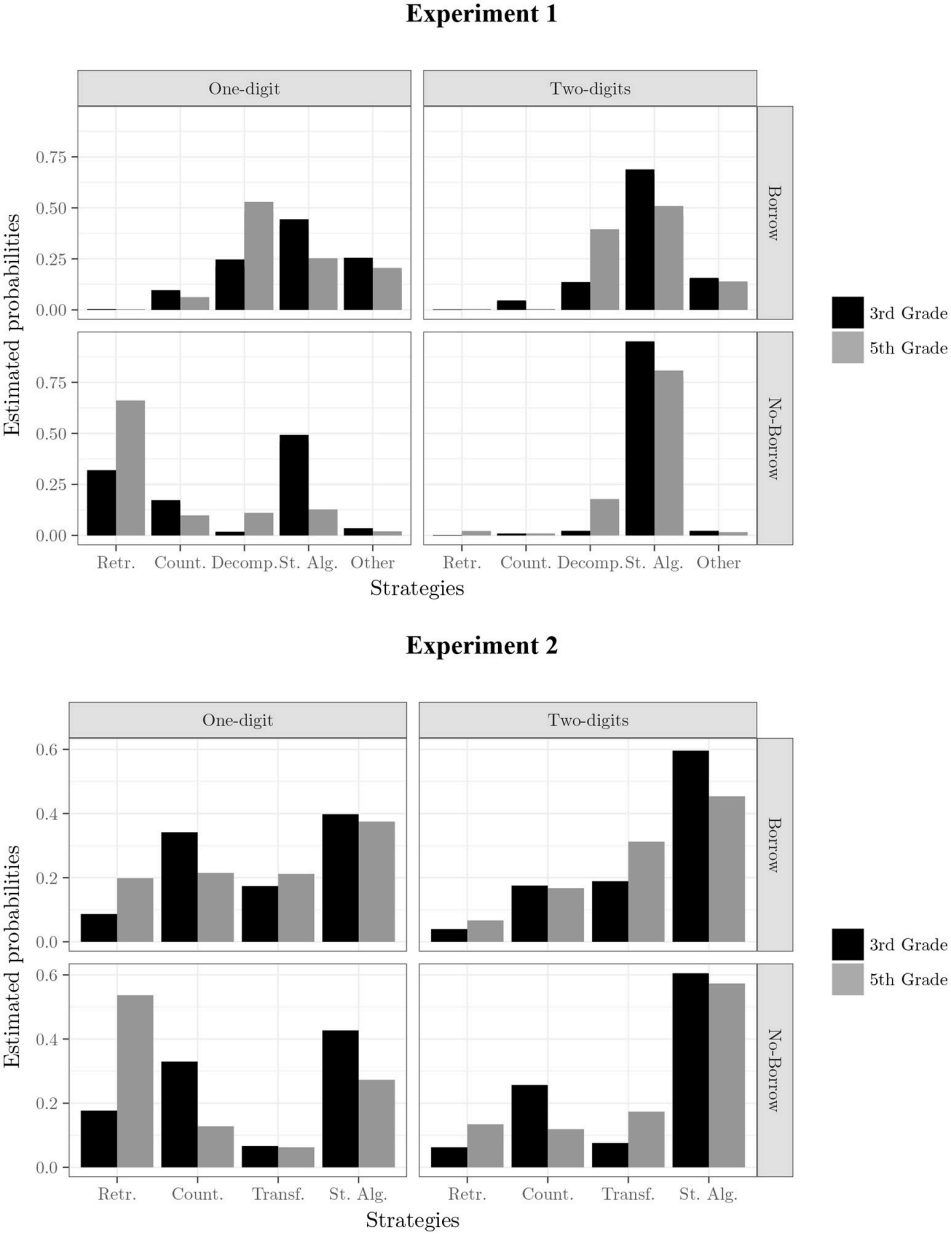
Principal effects of the best model for prediction of strategy choice in both Experiments. Figures show a representation of the estimated probability of strategy choice according the interaction of grade^*^borrow^*^complexity. Presentation format is not reported in the figure because it enters in the models as additive factor. Retr., retrieval; Count, counting; Decomp, left-to-right procedure; St. Alg, right-to-left procedure; Other, confusing reporting.

In the first step, which involved considering additive effects only (comparable to a main effects model), including all four predictors, a positive ΔBIC value of 3,364 was found, indicating that it was a significantly better fit than the null model. This finding indicates that all four predictors influenced strategy selection. Subsequently, inclusions of two- and three-way interactions improved the overall model fit. Following this procedure, the best-fitting model was *M11* (see Table 3), which included the interaction of three factors, that is borrow × grade × complexity and an additive effect of presentation format. Comparing the ΔBIC values, we found that M11 explained the data more than a million times (*Log BF* = 14) better than any of the other models. The interactive portion of the M11 model is represented in Figure 2 (upper panel), which shows the estimated probability for each strategy as a function of each combination of grade, complexity, and condition (borrow vs. no-borrow). The three-way interaction reflects the influence of the older children's greater experience and reveals clear differences in strategy choice across the problem features. In particular, the strategy used most frequently was the right-to-left procedure (i.e., St. Alg. In Figure 2): It was used on more problems than other strategies by both third- and fifth-graders on two-digit problems, but the younger children tended to use this procedural strategy even more often than the older children.

Other differences in strategy choice were found that were also related to grade. For example, retrieval was reported more frequently by fifth- than by third-graders on the simpler problems, that is, on no-borrow problems with a single-digit subtrahend (e.g., 57– 6). Both counting and decomposition strategies were generally reported less frequently than the right-to-left algorithm, except on one-digit borrow problems for fifth graders, where this strategy was the most frequent. Counting was reported somewhat more often by third- than by fifth-grade children, for all problems except the two-digit no-borrow problems. The left-to-right decomposition strategy was reported more often by fifth- than by third-grade children, specifically on problems with two-digit subtrahends, although the differences were modest. Finally, children's strategy reports were more likely to include a mixture of strategies (i.e., Other in Figure 2) borrow problems, especially one-digit ones. In summary, across grades, children showed a pattern of shifting from counting to retrieval strategies on the easier problems, that is, those with one-digit subtrahends, and a similar, but smaller shift toward left-to-right strategies on the harder problems, especially in the borrow condition. As Figure 2 (upper panel) highlights, the presence or absence of borrowing as well as the complexity of the subtrahend interacted to determine which strategy children selected on specific problems, suggesting that they were influenced by these factors as they chose which strategies to implement.

An additive effect of presentation format was observed, showing that this feature did not interact with problem characteristics in influencing children's strategy choices: the difference in the frequency use according the vertical or horizontal presentation format was small but consistent across other combinations of predictors. This is an interesting and novel finding, because it indicates that strategy choice can be influenced both by factors inherent to the solution process (i.e., problem complexity) and by features of the visual display (i.e., format).

## Experiment 2

In Experiment 2 we explored patterns of strategies chosen by children using an extended forced-choice condition. As in Experiment 1, children performed the same two tasks—the paper-and-pencil tasks assessing arithmetical achievement and the computerized mental subtraction task—with the only difference related to the collection of the strategy used. In this experiment, children were asked to choose the strategies used among a repertoire of alternatives based on the solution procedures resulted from Experiment 1. The goals of this experiment were to (a) replicate the results emerged in Experiment 1 and (b) determine whether the pattern of factors that emerged in the multinomial analysis is generalizable to data collected via another method of assessing strategy choice.

## Methods

### Participants

Participants included 175 children: 88 in third grade (47 boys, 41 girls) with a mean age of 100.2 months (*SD* = 3.6; range = 93–107.5 months), and 87 in fifth-grade (39 boys, 48 girls) with a mean age of 124.7 months (*SD* = 4.3; range = 109–140.5 months) who were attending Italian urban state schools. Parental consent was obtained. Children with special educational needs, specific learning disorders, intellectual disabilities, or neurological/genetic disorders, as indicated by their teachers, were not included in the study.

### Procedure

The design and procedure exactly replicated those of Experiment 1, with the sole exception being the way children strategy choices were collected. Children were asked to indicate how they had solved each problem by choosing one of four strategies (counting, retrieval, left-to-right decomposition, or right-to-left algorithm), which were explained with examples at the beginning of the individual session. Thus, in the present research, after completing each operation, participants were asked to indicate which out of four strategies they had used to solve each problem.

Children were randomly assigned to either the no-borrow or borrow condition, such that there were 89 children (45 boys, 44 girls; 48 third- and 41 fifth-graders) in the no-borrow condition, and 86 (41 boys, 45 girls; 40 third-, and 46 fifth-graders) in the borrow condition.

## Results

### Arithmetical academic achievement

Performance on the arithmetic achievement tasks was analyzed in 2 (grade) × 2 (condition: borrow, no-borrow) ANOVAs. No differences were observed neither in relation to the grade nor to the assigned condition (see Supplementary Material).

### Accuracy and response times

As in Experiment 1, the descriptive statistics for performance on the multi-digit subtraction task are shown in Table 1 (lower panel). Both percentage of correct responses and correct mean latency were examined with separate 2 (complexity: one- vs. two-digit numbers in the subtrahend) by 2 (format: horizontal, vertical presentation) × 2 (grade: 3, 5), × 2 (condition: borrow, no-borrow) mixed ANOVAs, with repeated measures on the first two factors. The results of these analyses are shown in Table 5.

**Table 5:** Results of the mixed-design 2 × 2 × 2 × 2 ANOVAs for the accuracy and RTs, with grade (third and fifth grade) and condition (absence or presence of borrowing procedure) as the between-participants factors, and complexity (single or double-digit subtrahend) and format (horizontal or vertical presentation) as repeated measures (Experiment 2).

	**Accuracy**				**Reaction times**		
	***df***	***F***	***P***	***LogBF***	***F***	***P***	***LogBF***
Grade (G)	1,171	9.95^**^	0.002	14.75	17.86^**^	< 0.0001	25.98
Condition (Cond)	1,171	45.64^**^	< 0.0001	61.95	78.99^**^	< 0.0001	99.52
Complexity (C)	1,171	104.24^**^	< 0.0001	123.88	397.57^**^	< 0.0001	315.29
Format (F)	1,171	0.27	0.604	0.31	3.47	0.064	5.25
G^*^Cond	1,171	2.24	0.136	3.41	1.99	0.160	3.04
G^*^C	1,171	3.50	0.063	5.29	8.63^**^	0.004	12.91
G^*^F	1,171	0.04	0.841	11.77	0.41	0.521	1.53
Cond^*^C	1,171	7.87^**^	0.006	0.06	1.01	0.318	0.64
Cond^*^F	1,171	2.89	0.091	4.36	1.36	0.245	2.08
C^*^PF	1,171	0.99	0.322	1.52	26.18^**^	< 0.0001	37.15
G^*^Cond^*^C	1,171	0.66	0.419	1.00	0.03	0.854	0.05
G^*^Cond^*^F	1,171	1.53	0.217	2.34	0.01	0.921	0.02
G^*^C^*^F	1,171	0.01	0.908	0.02	0.53	0.466	0.82
Cond^*^C^*^F	1,171	2.10	0.150	3.20	6.62^*^	0.011	9.97
G^*^Cond^*^C^*^F	1,171	0.04	0.843	0.06	0.01	0.916	0.02

*Strategy, type of strategy (i.e., counting, retrieval, decomposition, and written calculation); Grade: 3rd and 5th grade; Complexity, single or double-digit subtrahend; Condition (absence or presence of borrowing procedure); Pres.form, vertical and horizontal presentation format*.

Regarding the accuracy, the main effect of grade was significant, showing that younger children performed significantly worse than the older ones (73 vs. 82%). Consistent with Experiment 1, the effects of condition and complexity were significant: No-borrow problems were easier to solve than borrow problems (87 vs. 68%), and problems with one-digit subtrahends were easier to solve than those with two-digit subtrahends (85 vs. 70%). In contrast to Experiment 1, there were no significant effects of format. The only significant interaction was condition × complexity which confirmed that difference between borrow and no-borrow problems was larger for more complex problems (i.e., two-digit: +24%; 58 vs. 82%: one-digit: +15%; 78 vs. 93%; *p*_*s*_ < 0.001).

Similar to the results for accuracy, there were significant main effects of grade, condition (borrow status), and complexity in the analysis of response time. Hence, third-graders were slower than fifth graders (15 vs. 11 s), children who solved borrow problems responded more slowly than those who solved no-borrow problems (17 vs. 9 s), and children solved problems with double-digit subtrahends more slowly than those with single-digit subtrahends (16 vs. 10 s). The interaction of grade x complexity was significant. At both age groups, children were faster to solve problems with one-digit than those with two-digits subtrahends (*p*s < 0.001), however this difference was larger for children in third grade than those in fifth grade (12 vs. 19 s for third graders and 9 vs. 14 s for fifth graders). As in Experiment 1, the complexity x format and the condition x complexity x format interactions were significant. In particular, children did not show any effects of format during the execution of no-borrow problems whereas when children solved borrow problems, they were faster in vertical than in horizontal format with two-digit problems (15 vs. 17 s, *p* < 0.001) and slower in vertical format than in horizontal format with single-digit problems (11 vs. 9 s, *p* < 0.001). The three-way interaction among condition, complexity and format is shown in Figure 1 (lower panel).

### Strategy choice

As in the previous experiment, we analyzed strategy choices in order to determine whether they varied with the same problem features as did strategy efficiency, regardless of whether those choices resulted in accurate performance (see Supplementary Materials for the observed frequency of the strategies). The descriptive data on strategy choices are reported in Table 3. As for Experiment 1, the number of children who reported using each strategy at least once varied significantly across ages for all four strategies. More older children reported retrieval than younger children (80 vs. 44%), χ^2^ (1, *N* = 175) = 24.38, *p* < 0.001, Cramer's phi = −0.373 whereas fewer older children reported counting than younger children (68 vs. 86%), χ^2^ (1, *N* = 175) = 8.53, *p* = 0.003, Cramer's phi = 0.221. For the more complex strategies, more older than younger children reported using the left-to-right strategy (68 vs. 48%), χ^2^ (1, *N* = 175) = 7.23, *p* < 0.007, Cramer's phi = −0.203. Finally, although a majority of children in both grades reported using the right-to-left algorithm, more younger than older children used the strategy at least once (98 vs. 90%), χ^2^ (1, *N* = 175) = 4.84, *p* = 0.023, Cramer's phi = 0.166. Thus, in line with the previous experiment, the overall comparison of strategy repertoire across grade shows changes as a function of children's expertise. In the next paragraph, we analyze the patterns of strategy selection according to problem features.

Multinomial models and a model-selection strategy were used to analyze strategy choices in relation to problem features and grade on a trial-by-trial basis (175 × 32 = 5600 trials), as described in Experiment 1. Details of the multinomial process and the indexes that guided the model selection are reported in Table 6.

**Table 6:** Model comparison for strategy choice in Experiment 2.

	***BIC***	***Δ_*BIC*_***	***Log BF***	**Model**
M0	1453	0		1
M1	401	1052	16.20	pres.form + condition + grade + complexity
M2	385	1068	8.32	complexity + condition ^*^ grade + pres.form
M3	391	1061	11.55	pres.form + condition + grade ^*^ complexity
M4	400	1053	15.89	grade ^*^ pres.form + condition + complexity
M5	398	1055	14.59	pres.form + grade + complexity ^*^ condition
M6	406	1047	18.75	grade + condition ^*^ pres.form + complexity
M7	402	1051	16.66	condition + grade + pres.form ^*^ complexity
M8	397	1056	14.27	grade ^*^ pres.form + condition ^*^ complexity
M9	397	1056	14.09	condition ^*^ pres.form + complexity ^*^ grade
M10	386	1067	8.78	complexity ^*^ pres.form + grade ^*^ condition
M11	368	1084		condition ^*^ grade ^*^ complexity + pres.form
M12	396	1057	13.70	complexity + condition ^*^ pres.form ^*^ grade
M13	399	1054	15.29	condition + pres.form ^*^ grade ^*^ complexity
M14	409	1043	20.56	grade + condition ^*^ complexity ^*^ pres.form

*BIC, Bayesian Information Criterion; Δ_BIC_, BIC difference with respect to the null model (M0); LogBF, approximated bayes factor respect to the best model (M11) calculated through the relative likelihood [i.e., (Δ_BIC_/2)]. The higher the Δ_BIC_ the better the model. Strategy, type of strategy (i.e., counting, retrieval, decomposition, and written calculation); Grade, 3rd and 5th grade; Complexity: single or double-digit subtrahend; Condition (absence or presence of borrowing procedure); Pres.form, vertical and horizontal presentation format*.

These analyses precisely confirmed the former results: the best-fitting model was *M11*, which included the interaction of the three same factors, borrow x grade x complexity, and an additive effect of presentation format. Comparing the ΔBIC values, we found that M11 explained the data more than 2,900 times (*Log BF* = 8) better than any of the other models. Figure 2 (lower panel) shows the estimated probability of this model for each strategy as a function of each combination of grade, complexity, and condition (borrow vs. no-borrow). The overall pattern confirmed the strategy used most frequently was the right-to-left procedure: It was used on more problems than other strategies by both third-graders on all four types of problems and by fifth-graders on all except single-digit no-borrow problems, which were very frequently solved with retrieval. Retrieval was reported more frequently by fifth- than by third-graders on all problems, especially the simpler ones, and both counting and decomposition strategies were generally reported less frequently than the right-to-left algorithm. Compared to Experiment 1, counting was generally used more often, especially by third-grade children, for all problems except the hardest (i.e., two-digit borrow problems). The left-to-right decomposition strategy was reported somewhat more often by fifth- than by third-grade children, specifically on problems with two-digit subtrahends, although it was less used compared to Experiment 1. These analyses confirmed that the presence or absence of borrowing as well as the complexity of the subtrahend interacted with children's expertise to determine which strategy they selected on a specific problem.

This pattern of results strengthens the secondary role of presentation format that seems not to directly influence children's strategy choices: small differences in the frequency use emerged according the vertical or horizontal format and, above all, consistent across other combinations of predictors.

## Discussion

Children use a variety of strategies to solve mathematical problems (e.g., Barrouillet et al., 2008). Their strategy repertoire is assumed to reflect an integrated network of conceptual and procedural knowledge that allows them to decide how to perform a strategy, when to use it, and why (Hiebert and Lefevre, 1986; Bisanz and LeFevre, 1990). The goal of the present research was to explore key factors that influence children's strategy choices on multi-digit subtraction problems and to directly compare two different methods for assessing children's strategy choices. To achieve this end, two different experiments were conducted on similar cohorts of third- and fifth-grade children: In the first experiment, strategy selection was investigated by means of a *free-choice* (verbal self-report) paradigm, whereas in the second study a *discrete-choice* approach was implemented. Problem features, such as complexity (i.e., whether there were one- or two-digit subtrahends) and whether the solution crossed a decade boundary (i.e., required a borrow operation) were manipulated, in addition to presentation format (i.e., horizontal vs. vertical alignment). Classical statistical analyses were applied to children's performance (i.e., accuracy and response times), and multinomial models were used to analyze strategy choices in relation to problem features and grade on a trial-by-trial basis.

Analyses of accuracy and response times in both experiments showed typical age-related improvement in performance: Fifth-grade children solved problems more quickly and accurately than third-grade children. Children's accuracy was sensitive to problem features that influence the difficulty of the problem, specifically, children assigned to the borrow condition correctly solved fewer problems than children assigned to the no-borrow condition and both groups were less accurate in solving problems with a double-digit subtrahend. A comparison of the results of the two studies revealed a discrepancy related to the contextual feature: In the first experiment children's performance was influenced by the presentation format only when they had to solve the hardest problems (vertical presentation improved correct responses); whereas, in the second experiment, children's accuracy was not sensitive to presentation format. Children's latencies, in contrast, were related to all of the problem features, and showed the same pattern of significant effects in both experiments. Increased complexity (both in terms of borrowing procedure and subtrahend size) slowed problem execution.

Presentation format also influenced solution latencies in relation to problem difficulty: Children were faster to correctly solve double-digit borrow problems presented in columns than in rows, whereas the reverse pattern was found for single-digit problems. The differential efficiency of performance shown on correct latencies (i.e., for borrow problems, children were faster in horizontal format for one-digit problems such as 73–5 but faster with vertical format for two-digit problems such as 43–29) suggests that choices were not strategic, per se, but were driven more directly by problem format. This conclusion is consistent with the absence of any interactions between presentation format and either grade or complexity on strategy choice. Other research has suggested that different working memory resources may be implicated as a function of presentation format (e.g., Trbovich and LeFevre, 2003; Caviola et al., 2012). Thus, manipulation of presentation format may influence strategy choices independently of factors that are related to expertise or problem complexity.

The increased level of performance with age corresponds to similar patterns found in previous research (see Campbell, 2005; Cohen Kadosh and Dowker, 2015 for a general overviews), such that children's performance on complex subtraction problems is linked to their level of experience (i.e., school grade) and to variability in problem features that reflect computational processes (Imbo and Vandierendonck, 2007c; Lemaire and Calliès, 2009). Novel results were obtained for presentation format where effects occurred only on borrow problems and varied with complexity. These patterns were further qualified by the analyses of strategy choice, as described below. Further research on the relations between superficial features and those tied directly to computational demands may have important implications for understanding children's solution processes on complex problems. For example, it would be interesting to better understand how different combinations of characteristics, such as problems presented in other familiar formats (e.g., auditory; Noël et al., 1997; LeFevre et al., 2001), may also influence accuracy and response times.

The second novel and interesting set of results concerns children's strategy choice. No previous research defined in detail the full range of strategies used by children to solve complex subtraction problems. In both experiments, we analyzed strategy choices using multinomial modeling in which all factors, that is, expertise (i.e., grade), complexity of problem, condition (i.e., borrow vs. no-borrow) and presentation format were included as predictors. Interestingly, the best-fitting model was the same in both experiments and included a three-way interaction of grade, condition, and complexity, and an additive effect of presentation format. First, consider problems with one-digit subtrahends. We observed in both studies that fifth-grade children choose retrieval more than third graders on no-borrow problems (e.g., 78–5) whereas third-grade children were more likely to choose the standard algorithm. In contrast, third-grade children chose counting more often than fifth graders on both borrow (e.g., 73–5) and no-borrow problems (e.g., 89–7). These patterns for single-digit subtractions show a shift from less- to more-sophisticated strategies with expertise (i.e., more retrieval, less counting), accompanied by a higher reliance on algorithmic solutions by the younger children.

For the more difficult problems with two-digit subtrahends, compared to fifth-graders, third graders chose counting more often on no-borrow problems (e.g., 68–41), and the standard algorithm more often on borrow problems (e.g., 43–29). Compared to third-graders, fifth graders more often chose decompositions for both borrow and no-borrow problems. Again, these patterns of strategy choice, emerged in both studies, indicate that older children, relied more on strategies that were efficient (i.e., less use of counting) and reflected their superior conceptual understanding (i.e., more use of decompositions). It is worth to remember that these differences which emerged in strategies selection may reflect a schooling or recency effect (Lemaire and Brun, 2018): third graders may be more likely to choose a standard (written) algorithm solution because it is a strategy that they recently learnt at school (it is taught during the second and third grades in Italy), whereas older children can rely on more efficient strategies (i.e., decomposition) linked to a better mastery of basic arithmetic knowledge.

At a more general level, multinomial modeling of strategy choices confirmed the importance of some key influences on children's strategy choices for subtraction. The presence or absence of borrowing, the value of the subtrahend, and presentation format all influenced which strategy was adopted to solve the problems. Moreover, in line with our expectations, children varied in their strategy repertoires and their use of those strategies according to their level of experience and in relation to problem complexity. These findings extend results reported in previous studies, encompassing a wider range of subtraction problems. Previous research on simple addition problems indicated that children tend to shift from counting (an inefficient procedural strategy) to more efficient memory-based retrieval with increased age (Widaman et al., 1992; Lemaire and Siegler, 1995; Geary, 2004; Reed et al., 2015). The present results support a similar pattern for complex subtraction, but show that shifts are related to problem features and that there is also considerable persistence in strategy availability with development from grades three to five. Taken together, these outcomes support Siegler's overlapping waves model (1996), at least for the retrieval vs. non-retrieval strategies: Children do not simply use a particular strategy until a better one is available, instead they have many strategies at once and it is frequency of use that changes across development (Lemaire and Siegler, 1995).

In previous research, children also showed developmental and educational changes in the use of left-to-right and right-to-left strategies (Fuson et al., 1988; Geary et al., 2004). Both the methods we used to assess strategy choice showed that children have a wide repertoire of strategies that overlapped from third- to fifth-grade, and that, although strategy choice changed with grade, it also depended heavily on problem features. Thus, the present study was consistent with the findings of persistent diverse strategy use across expertise, a finding observed even among adults solving simple arithmetic problems (e.g., LeFevre et al., 1996; Barrouillet et al., 2008), and extended the conclusion that children do not use a single strategy to solve two-digit subtraction problems (Lemaire and Calliès, 2009). Thus, our findings replicated similar patterns from previous studies and extended the overlapping waves model to a wider repertoire of strategies. In fact, these four strategy categories were often used in previous studies and account well for data observed in adults (e.g. Campbell and Xue, 2001; Campbell and Austin, 2002; LeFevre et al., 2006) whereas all of them were never considered together before in a developmental sample.

Finally, the present research shows the validity of two different self-report methods for assessing strategy choice: Both *free-choice* and *discrete-choice* approaches provided valuable information about strategy repertoire and strategy choices. Another important contribution of the present work is the use of a novel analysis of categorical data on strategy choices on a problem-by-problem basis. Together, the combination of self-report method and categorical analyses of those self-reports allowed us to document developmental changes in relation to different problem features that are known to influence strategy efficiency. Previously, the use of strategies has often been examined in terms of strategy efficiency and adaptivity (Lemaire et al., 2000), which refers to the speed and accuracy with which strategies are implemented: A multilevel modeling approach extends the analyses of these aspects from an individual to an item level. Future studies may apply this approach which allows a sufficient amount of data for establishing temporal and accuracy characteristics of the strategies in a reliable way (Luwel et al., 2009).

As always, this research had limitations. First, as noted by Lemaire and Brun (2018), allowing students to have full choice of strategies does not allow an unbiased investigation of strategy execution and efficiency and so future studies should specifically address this limitation. Second, further research should explore how individual differences in cognitive resources can differently affect the pattern of strategies that students select and apply to different types of problems, maybe also including both simple and complex problems in a within subject design. For example, researchers have shown that children with mathematical difficulties distribute working memory recourses differently than do their typically-developing peers (Mammarella et al., 2013a,b). Future research should address this important issue because it has clear implications for scenarios outside the experimental setting, such as in teaching decisions (e.g., when teachers have to choose whether to focus on practice or on exploration and flexibility; Imbo and LeFevre, 2009), and in the clinical setting (e.g., for the development of effective intervention programs; Caviola et al., 2016). It is generally assumed that children experiencing mathematical learning difficulties find it difficult to use both retrieval and right-to-left strategies (see Geary, 2004, for a review). But a more in-depth knowledge of which strategies prove more efficient in relation to a problem's complexity and an individual's resources might help to improve such children's mathematical achievement and may be beneficial in the design of appropriate diagnostic tools and educational interventions. Finally, it is important to conduct cross-cultural studies to understand how cultural and schooling effects may influence strategy selection for children of various ages (Imbo and LeFevre, 2009, 2011).

In brief, the present research showed that there is great variability in strategy selection in complex subtraction problems and revealed important effects of grade, problem complexity, and presentation format on how participants solve complex arithmetic problems, both in terms of performance and in choice of strategies. We found that problem features influence performance, either because these physical qualities compromise the efficiency of strategies that they usually applied in mental calculation or because one or more of these features directly influences strategy choice.

## Ethics statement

The protocol was approved by the Ethics Committee of the University of Padova. Consent forms were sent to the parents. If the parents agreed to participate, they were sent them forms to fill in and their children were examined at their respective schools. Children generally reported the tasks within the realm of what they normally do in the school day.

## Author contributions

SC and IM developed the study concept. SC organized and supervised data collection. MP conducted the statistical analyses. SC and J-AL drafted the manuscript. IM and J-AL provided critical revisions. All authors approved the final version of the manuscript for submission.

### Conflict of interest statement

The authors declare that the research was conducted in the absence of any commercial or financial relationships that could be construed as a potential conflict of interest.
