# Supplementary material for: Children's Strategy Choices on Complex Subtraction Problems: Individual Differences and Developmental Changes
Source: Front Psychol. 2018 Jul 17;9:1209. doi: 10.3389/fpsyg.2018.01209 (PMC6057409; doi:10.3389/fpsyg.2018.01209)
Supplement: Supplementary file 1 [file Table_1.DOCX]

**Supplementary material**

Children’s performance on the arithmetical academic achievement in Experiment 1 and 2 respectively. Measures expressed in z-scores of correct responses (M=mean; SD=standard deviations), and results of ANOVAs.

| **Experiment 1** | | | | | | | | |
| --- | --- | --- | --- | --- | --- | --- | --- | --- |
|  | **Third grade** | | **Fifth grade** | | **Statistics** | | | |
|  | *M* | *SD* | *M* | *SD* | *df* | *F* | *p* | *η²* |
| **Written calculations** | .35 | 0.86 | .21 | 0.59 | 1,153 | 1.47 | .227 | .009 |
| **Mental calculation** | -.10 | 0.92 | .02 | 0.79 | 1,153 | .86 | .355 | .006 |
|  | **No-Borrow** | | **Borrow** | |  |  |  |  |
|  | *M* | *SD* | *M* | *SD* | *df* | *F* | *p* | *η²* |
| **Written calculations** | .34 | 0.74 | .23 | 0.73 | 1,153 | .75 | .389 | .005 |
| **Mental**  **calculation** | .03 | 0.76 | -.10 | 0.94 | 1,153 | .89 | .346 | .006 |

| **Experiment 2** | | | | | | | | |
| --- | --- | --- | --- | --- | --- | --- | --- | --- |
|  | **Third grade** | | **Fifth grade** | | **Statistics** | | | |
|  | *M* | *SD* | *M* | *SD* | *df* | *F* | *p* | *η²* |
| **Written calculations** | -.41 | 0.99 | -.19 | 1.05 | 1,173 | 1.81 | .181 | .010 |
| **Mental calculation** | -.30 | 1.13 | -.15 | 1.07 | 1,173 | .83 | .364 | .005 |
|  | **No-Borrow** | | **Borrow** | |  |  |  |  |
|  | *M* | *SD* | *M* | *SD* | *df* | *F* | *p* | *η²* |
| **Written calculations** | -.37 | 1.09 | -.23 | 0.96 | 1,173 | .77 | .381 | .004 |
| **Mental**  **calculation** | -.31 | 1.17 | -.14 | 1.02 | 1,173 | .94 | .334 | .005 |

Set of problems used in the two experimental contidions.

| No borrow problems | | | |
| --- | --- | --- | --- |
| Single digit subtrahend | | Double digit subtrahend | |
| 95 – 1 | 78 – 5 | 28 – 15 | 57 – 32 |
| 36 – 2 | 29 – 6 | 27 – 16 | 79 – 37 |
| 67 – 2 | 97 – 6 | 38 – 17 | 68 – 41 |
| 56 – 3 | 28 – 7 | 39 – 18 | 59 – 46 |
| 75 – 3 | 89 – 7 | 45 – 21 | 96 – 52 |
| 58 – 4 | 48 – 8 | 49 – 23 | 86 – 54 |
| 87 – 4 | 69 – 8 | 65 – 24 | 87 – 63 |
| 47 – 5 | 39 – 9 | 77 – 25 | 98 – 68 |

| Borrow problems | | | |
| --- | --- | --- | --- |
| Single digit subtrahend | | Double digit subtrahend | |
| 73 – 5 | 27 – 8 | 30 – 16 | 41 – 35 |
| 91 – 5 | 41 – 8 | 26 – 17 | 52 – 37 |
| 32 – 6 | 54 – 8 | 37 – 18 | 74 – 46 |
| 53 – 6 | 75 – 8 | 23 – 19 | 56 – 49 |
| 45 – 7 | 26 – 9 | 64 – 25 | 85 – 58 |
| 62 – 7 | 38 – 9 | 75 - 27 | 92 – 59 |
| 84 – 7 | 68 – 9 | 61 – 28 | 83 – 67 |
| 96 – 7 | 87 – 9 | 43 – 29 | 90 – 68 |
